# Heterozygous premature termination in zinc-finger domain of Krüppel-like factor 2 gene associates with dysregulated immunity

**DOI:** 10.3389/fimmu.2022.819929

**Published:** 2022-11-18

**Authors:** Nora Pernaa, Salla Keskitalo, Iftekhar Chowdhury, Antti Nissinen, Virpi Glumoff, Riikka Keski-Filppula, Juhani Junttila, Kari K. Eklund, Wenny Santaniemi, Sanna Siitonen, Mikko RJ. Seppänen, Paula Vähäsalo, Markku Varjosalo, Pirjo Åström, Timo Hautala

**Affiliations:** ^1^ Research Unit of Biomedicine, University of Oulu, Oulu, Finland; ^2^ Molecular Systems Biology Group, Institute of Biotechnology, University of Helsinki, Helsinki, Finland; ^3^ PEDEGO Research Unit, University of Oulu, Oulu, Finland; ^4^ Department of Clinical Genetics, Oulu University Hospital, Oulu, Finland; ^5^ Medical Research Center Oulu, University of Oulu and Oulu University Hospital, Oulu, Finland; ^6^ Research Unit of Internal Medicine, Medical Research Center Oulu, University of Oulu and Oulu University Hospital, Oulu, Finland; ^7^ Department of Rheumatology, Inflammation Center, University of Helsinki and Helsinki University Hospital and Orton Orthopedic Hospital, Helsinki, Finland; ^8^ Oulun University Hospital and Research Unit of Biomedicine, University of Oulu, Oulu, Finland; ^9^ Department of Clinical Chemistry, University of Helsinki and HUSLAB, Helsinki University Hospital, Helsinki, Finland; ^10^ Rare Disease Center and Pediatric Research Center, Children and Adolescents; Adult Immunodeficiency Unit, Inflammation Center, University of Helsinki and Helsinki University Hospital, Helsinki, Finland; ^11^ Department of Pediatrics, Oulu University Hospital, Oulu, Finland; ^12^ Infectious Diseases, Oulu University Hospital and Research Unit of Biomedicine, University of Oulu, Oulu, Finland

**Keywords:** immune dysregulation, autoimmunity, inborn error in immunity, lymphocyte trafficking, maturation, juvenile idiopathic arthritis, rheumatoid arthritis

## Abstract

Krüppel-like factor 2 (KLF2) is a transcription factor with significant roles in development, maturation, differentiation, and proliferation of several cell types. In immune cells, KLF2 regulates maturation and trafficking of lymphocytes and monocytes. KLF2 participates in regulation of the nuclear factor kappa-light-chain-enhancer of activated B cells (NF-κB) pathway. Although pulmonary arterial hypertension (PAH) related to KLF2 genetic variant has been suggested, genetic role of KLF2 associated with immune dysregulation has not been described. We identified a family whose members suffered from lymphopenia, autoimmunity, and malignancy. Whole exome sequencing revealed a KLF2 p.(Glu318Argfs*87) mutation disrupting the highly conserved zinc finger domain. We show a reduced amount of KLF2 protein, defective nuclear localization and altered protein-protein interactome. The phenotypically variable positive cases presented with B and T cell lymphopenia and abnormalities in B and T cell maturation including low naive T cell counts and low CD27^+^IgD^-^IgM^-^ switched memory B cells. KLF2 target gene (CD62L) expression was affected. Although the percentage of (CD25^+^FOXP3^+^, CD25^+^CD127^-^) regulatory T cells (Treg) was high, the naive Treg cells (CD45RA^+^) were absent. Serum IgG1 levels were low and findings in one case were consistent with common variable immunodeficiency (CVID). Transcription of NF-κβ pathway genes and p65/RelA phosphorylation were not significantly affected. Inflammasome activity, transcription of genes related with JAK/STAT pathway and interferon signature were also comparable to controls. Evidence of PAH was not found. In conclusion, KLF2 variant may be associated with familial immune dysregulation. Although the KLF2 deficient family members in our study suffered from lymphopenia, autoimmunity or malignancy, additional study cohorts are required to confirm our observations.

## 1 Introduction

Krüppel-like factors (KLF) are members of the zinc finger transcription factor family with diverse biological roles including the regulation of quiescence, proliferation, differentiation, and trafficking of various cell types (1–3). In immune cells, KLF2 activity is needed for B cell, T cell and monocyte development and migration (3). For example, regulation of inflammatory responses by the nuclear factor kappa-light-chain-enhancer of activated B cells (NF-κB) pathway, is partly dependent on KLF2 activity (4, 5). KLF2 expression in endothelial cells is suppressed by pro-inflammatory cytokines such as tumor necrosis factor alpha (TNF-α) and interleukin (IL)-1β (6). KLF2 is also involved in the regulation of immunological tolerance; for example, forkhead box P3 (FOXP3) expression in induced regulatory T (iTreg) cells may depend on KLF2 activity (7). Importantly, KLF2 controls the expression of trafficking molecules such as L-selectin (CD62L), C-C Motif Chemokine Receptor 7 (CCR7) and Sphingosine-1-phosphate receptor 1 (S1PR1) during lymphocyte maturation. KLF2 deficiency in experimental mouse models leads to upregulation of T cell activation markers and to loss of peripheral T cells (8, 9). Homozygous KLF2^-/-^ mouse is embryonically lethal (10). Hemizygous KLF2+/- mice exhibit, for example, defective macrophage function and abnormal inflammatory responses (11) (4, 12) and myeloid specific KLF2 deficiency exacerbates neuroinflammation in murine multiple sclerosis model (13). Diverse effects of KLF2 deficiency on immune cells are carefully reviewed by Wittner and Schuh (3).

KLF2 is thought to have a role in pathogenesis of autoimmunity (2) and has a protective role in the K/BxN arthritis model in mice (14). In human disease, somatic mutations with variable variant allele frequencies in the *KLF2* are frequently found in malignant B cell clones in splenic marginal zone lymphoma (SMZL) (15–18). Although a KLF2 mutation was suggested to be associated with pulmonary arterial hypertension (PAH) (19), an inborn error in immunity (IEI) associated with KLF2 gene has not been reported. We identified a family presenting with lymphopenia, juvenile idiopathic arthritis, rheumatoid arthritis, and malignancy. Exome sequencing revealed a heterozygous mutation causing a shift in the reading frame within the highly conserved KLF2 zinc finger domain. We studied the biological consequences as well as clinical and immunological manifestations associated with the variant. In this report, we describe novel features of immune dysregulation associated with a KLF2 mutation.

## 2 Materials and methods

### 2.1 Patients

**Figure 1:**
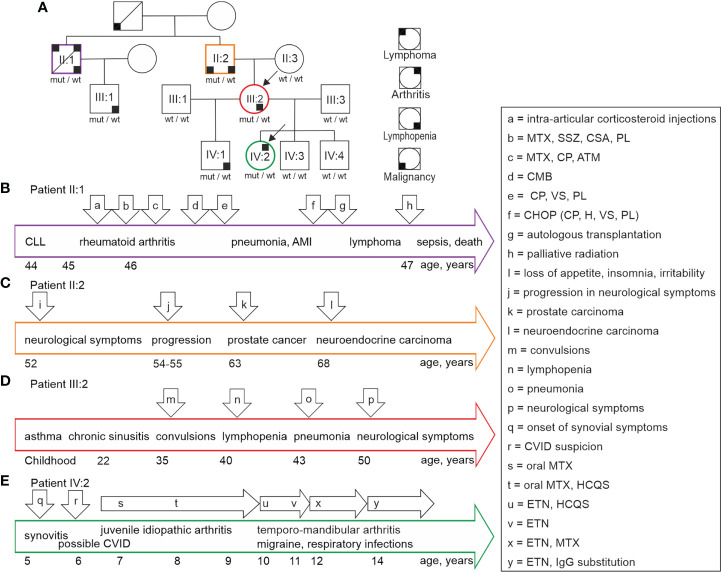
**(A)** Family tree illustrates the individuals with KLF2 c.951dup p.(Glu318Argfs*87) mutation. Black arrows point at the index patient IV:2 diagnosed with idiopathic juvenile arthritis and the index patient III:2 with lymphopenia of unknown origin. The variant positive family members suffer from lymphopenia, arthritis, lymphoma, and malignancies marked with small black boxes in four alternative corners, each indicating a different condition. Summary and timeframe (years) of clinical events in patients II:1 **(B)**, II:2 **(C)**, III:2 **(D)** and IV:2 **(E)** is shown. The major health events and medications are indicated by arrows (a to x). The corresponding health events are explained in detail in chapter 2.1. CLL, Chronic lymphocytic leukemia; MTX, methotrexate; SSZ, sulfasalazine; CSA, cyclosporine; PL, prednisolone; ATM, sodium aurothiomalate; CP, cyclophosphamide; CMB, chlorambucil; VS, vincristine sulfate; H, hydroxydaunorubicin; CVID, common variable immunodeficiency; HCQS, hydroxychloroquine; ETN, etanercept.

We identified a family with unusual clinical presentations (**Figure 1**; arrows a– x indicate events described for patients). The index members suffered from unexplained lymphopenia (III:2) and juvenile idiopathic arthritis in association with findings suggestive of common variable immunodeficiency-like state (IV:2). The other KLF2 variant positive family members (II:1, II:2, IV:1) presented with variable lymphopenia, neurological symptoms, autoimmunity, and malignant diseases as described in detail below. None of the variant negative family members (II:3, III:1, III:3, IV:3, IV:4) presented with any clinical or laboratory evidence of abnormal immunity. All family members gave their informed consent for this study. The study has been approved by the Ethics Committee of Oulu University Hospital.

#### 2.1.1 Patient I:1

This historical patient was born to non-consanguineous parents. According to aged relatives, he had been healthy without obvious infection susceptibility or signs of autoimmune diseases. He had experienced a sudden deterioration of his health at age 65. Subsequently, he received a leukemia diagnosis. Criteria for this condition are not available. The patient had died within two months of the diagnosis. At that time, no specific treatments had been available.

#### 2.1.2 Patient II:1

A previously healthy male had an elevated peripheral white blood cell (WBC) count (18.0×10^9^/L, normal 4.0-11.0×10^9^/L) in a routine health inspection at age 44. Subsequently he was diagnosed to suffer from chronic lymphocytic leukemia (CLL) without an immediate indication for medical treatments. The clonal CD19^+^ B cells were kappa^+^CD15^+^CD20^+^CD11c^+^CD23^+^CD25^+^. At age 45, he developed chronic synovitis affecting multiple small and large joints caused by seropositive rheumatoid arthritis. He was positive for rheumatoid factor (RF) (501 IU/L, normal <25), and negative for HLAB27 and antinuclear antibodies (ANA-ab). Antibodies against cyclic citrullinated peptides (CCP-ab) had not been analyzed.

The timeline of events is summarized in **Figure 1B**. The arthritis was very active, and he had received local corticosteroid injections (**Figure 1B**, a). Combinations of methotrexate, sulfasalazine, cyclosporine and prednisolone were ineffective to control the arthritis (**Figure 1B**, b). In addition, the combination of cyclophosphamide and methylprednisolone was also insufficient. Methotrexate, cyclosporine, and sodium aurothiomalate combination proved effective (**Figure 1A**, c). Although the symptoms alleviated, synovial erosions progressed in radiological evaluations.

The patient developed anemia (blood hemoglobin 108 g/L), an elevation in WBC (26.9×10^9^/L), and a decrease in serum immunoglobulin G (IgG) concentrations. 35% of his bone marrow aspirate were clonal B cells. Mild lymphadenopathy was observed, and spleen size was normal (14cm). The patient developed febrile episodes and muscle aches during chlorambucil treatment (**Figure 1B**, d). He started receiving fludarabine treatments mainly because of progression in the number and the size in lymph nodes; no response was observed. However, the patient responded to cyclophosphamide, vincristine, and prednisolone (**Figure 1B**, e). This was followed by anemia and low WBC. Bone marrow aspirate showed that 80% of cells displayed lymphatic origin with immature morphology. Megaloblastoid cells were also present. The findings were suggestive of myelodysplasia. The patient had an episode of severe pneumonia associated with acute myocardial infarction (AMI).

After recovery from pneumonia and AMI, the patient received cyclophosphamide, hydroxydaunorubicin, vincristine and prednisolone (CHOP) treatments (**Figure 1B**, f). In addition, he received carmustine, etoposide, Ara C and cyclophosphamide (BEAC) followed by autologous bone marrow transplantation (**Figure 1B**, g). Soon after, he was asymptomatic and able to return to his occupation. Six months later, he experienced a strong progression in the number and the size of lymph nodes. He received cladribine/rituximab with some response. Lymph node biopsies were consistent with diffuse large B-cell lymphoma (large cells CD20^+^CD15^-^CD30^-^, medium sized cells CD20^+^CD5^+^CD23^-^CD10^-^EMA^-^cyclin D1^-^kappa^-^lambda^-^). The patient received palliative radiation therapy for his lymphadenopathy (**Figure 1B**, h), and he suffered episodes of septic infections. Malignant cells infiltrated the bone marrow. The patient died at age 47 because of the malignant disease progression and aggressive infections associated with prolonged immunosuppression.

#### 2.1.3 Patient II:2

The patient was born to non-consanguineous parents. Timeline of clinical events is presented in **Figure 1C**. Childhood was unremarkable and there was no evidence of susceptibility to infections. He was healthy and active until age 52, when he developed a mental condition with loss of appetite, insomnia, and irritability (**Figure 1C**, i). His ability to concentrate and mental capacity deteriorated leading to inability to work. At that point, he was concluded to suffer from severe depression. Organic or infectious origin for the symptoms had not been considered. Due to progression of the symptoms, he required extended hospitalizations at psychiatric units. He received mirtazapine, olanzapine, lithium carbonate, and oxazepam medications.

Neurological evaluation was completed a year after the acute episode. The patient was hypomimic, psychologically reserved and slow in his movements. Sensation and tendon reflexes were normal, and he did not suffer from tremor or rigidity. Neuropsychological evaluation demonstrated impairment of attention and verbal memory. Electroencephalography (EEG), magnetic resonance imaging (MRI) and computed tomography (CT) of his brain were unremarkable. Blood and cerebrospinal fluid analyses for syphilis, HIV, sarcoidosis, borreliosis and tuberculosis were negative. In the follow up, the patient suffered from slow progression in the symptoms (**Figure 1C**, j). For example, he developed tremor, disturbance of balance and he required assistance in his daily tasks.

At age 63, he developed urinary incontinence caused by prostate hyperplasia. Subsequently, samples collected in transurethral resection of the prostate confirmed a local adenocarcinoma (**Figure 1C**, k) with 2% of cells showing malignancy (Gleason score 6). At follow up, the patient did not suffer from urological symptoms and blood prostate specific antigen (PSA) level remained low. In addition, several prostate biopsies were collected. In summary, no evidence suggesting the possibility of spread of the malignancy was found. At age 68, the patient developed acute abdominal pain and vomiting caused by obstructive gallstones. In association with the treatment of gallstones, a highly differentiated submucosal neuroendocrine carcinoma (**Figure 1C**, l) was found in his duodenum. This lesion did not relapse after surgical removal.

#### 2.1.4 Patient III:1

This male patient in his 30s has not suffered from infection susceptibility or any other condition consistent with immunological abnormality. Clinical evaluation and echocardiography findings did not support the possibility of PAH.

#### 2.1.5 Patient III:2

This patient had suffered from severe asthma since her childhood. Timeline of clinical events is presented in **Figure 1D**. She had repeated sinus infections and she suffered from migraine attacks. At age 30, she had a complicated delivery followed by severe and prolonged bleeding. At age 35, she had a sudden attack of unconsciousness and convulsions. Brain MRI was normal, and EEG was suggestive of localized left frontal and temporal non-epileptiform abnormalities (**Figure 1D**, m). Evidence of epilepsy was not found. At age 40, her lymphopenia was noted (**Figure 1D**, n), and she was sent to immunological analyses. At age 43, she was hospitalized because of pneumonia (**Figure 1D**, o). Bone marrow aspirate at age 50 was unremarkable. At age 50 the patient developed a transient episode of disturbed sensation on the right side of her face and oral cavity (**Figure 1D**, p). Clinical evaluation and echocardiography findings did not support the possibility of PAH.

#### 2.1.6 Patient IV:1

The patient had suffered from asthma since early childhood. He has no evidence of infection susceptibility, autoimmunity or autoinflammation. Lymphopenia was recognized at age 11. He was evaluated for his immunological properties at age 20. Clinical evaluation and echocardiography findings did not support the possibility of PAH.

#### 2.1.7 Patient IV:2

Timeline of clinical events is summarized in **Figure 1E**. This index patient had been healthy until the age of 5 when she complained of pain and stiffness in her knees especially in mornings (**Figure 1E**, q). Her WBC (2.7-5.1×10^9^/L; normal range 3.4-8.2×10^9^/L) and blood lymphocyte count (0.8-1.5×10^9^/L; normal range 1.2-3.5×10^9^/L) were slightly low or normal. Serum IgG (3.9-5.7 g/L; normal range 6.77-15 g/L), IgG1 (3.0 -3.7 g/L; normal range 4.9-11.4 g/L), and IgG3 0.13 – 0.21; normal range 0.2-1.1 g/L) concentrations were below normal. A low proportion of CD19^+^CD27^+^IgD^-^IgM^-^ switched memory B cells was recognized. These laboratory results obtained before immunosuppressive medications were suggestive of possible common variable immunodeficiency (CVID) (**Figure 1E**, r). Naproxen treatment given by a pediatric rheumatologist alleviated the symptoms, but this treatment caused stomachache. Two months later, arthritis was radiologically confirmed with MRI leading to juvenile idiopathic arthritis diagnosis (**Figure 1E**, s). At follow-up, the joint symptoms were active, and arthritis was found altogether in 5 joints (knee, ankle, and toes). C reactive protein (CRP) and sedimentation rate (ESR) were normal. Oral methotrexate (15 mg/m^2^/week) was started followed by an inadequate response and emesis (**Figure 1E**, s). Hydroxychloroquine was added to treatment with a satisfactory response (**Figure 1E**, t). However, the patient developed verrucosis on her hands. At the age of 9 years, the patient developed temporo-mandibular joint synovitis. Consequently, the oral methotrexate was changed to a subcutaneous product.

At age 10, migraine was diagnosed. At age 10 years and 6 months, subcutaneous methotrexate was discontinued because of low WBC. The patient started receiving a weekly 25 mg etanercept dose (**Figure 1E**, u). Hydroxychloroquine was stopped at age 11 years and 6 months (**Figure 1D**, v). At that time, the temporo-mandibular joint synovitis was in remission, but the patient continued to have low WBC. She developed episodes of eczema which were not obviously connected with medications. At age 12, methotrexate along with etanercept was started again because of active temporo-mandibular joint synovitis (**Figure 1E**, x).

At age 14, she suffered from repeated non-invasive respiratory tract infections. Chest CT and spirometry were unremarkable. The patient started receiving intravenous IgG (Privigen^®^) substitution (**Figure 1E**, y) with a good clinical response to respiratory infections. Methotrexate was stopped because of emesis. Currently, the juvenile idiopathic arthritis is inactive with etanercept (50 mg weekly) monotherapy.

### 2.2 Genetic analysis

Whole-exome sequencing (WES) (index cases III:2 and IV:2) was performed in a diagnostic laboratory (Centogene AG, Rostock, Germany). Other family members were analyzed for the KLF2 variant. In brief, RNA capture baits against approximately 60 Mb of the human coding exome (targeting >99% of regions in CCDS, RefSeq and Gencode databases) were used to enrich regions of interest from fragmented genomic DNA with the Agilent’s SureSelect Human All Exon V6 kit. The generated library was sequenced on an Illumina platform to obtain an average coverage depth of 100x. Typically, approximately 97% of the targeted bases were covered >10x. A Centogene in-house developed bioinformatics pipeline, including read alignment to GRCh37/hg19 genome assembly, variant calling and annotation, and comprehensive variant filtering, was applied. All disease-causing variants reported in the Human Gene Mutation Database (HMGD), in ClinVar or in CentoMD mutation database as well as all variants with minor allele frequency (MAF) of less than 1% in Genome Aggregation Database (gnomAD) were considered. Evaluation was focused on coding exons along with flanking +/-20 intronic bases. All pertinent inheritance patterns were considered. Family history and clinical information were used to evaluate the identified variants. All identified variants were evaluated with respect to their pathogenicity and causality. All variants related to the phenotype, except benign or likely benign variants, were reported.

Research variant (with potential reference to the described phenotype) was identified in the KLF2 gene. Targeted region of the KLF2 gene was analyzed by PCR and sequencing of both DNA strands of the entire coding region and the highly conserved exon-intron splice junctions. The reference sequence is KLF2: NM_016270.3.

### 2.3 Isolation and culture of cells

Peripheral blood mononuclear cells (PBMCs) were isolated by Ficoll-Paque gradient centrifugation (lithium heparin tubes) or from blood collected to BD Vacutainer cell preparation tubes (CPT, BD Biosciences). The cells were counted, and aliquots (90% FBS and 10% DMSO) were stored at -140°C for later use. In all experiments, age/sex matched control cells were used along with patient cells. In all *in vitro* flow cytometry experiments a fixable LIVE/DEAD Stain Kit was used to assess the viability of the cells (described below).

To isolate neutrophils, venous blood was collected into 9 ml lithium-heparin tubes, combined with equal volume of dextran solution (3% Dextran-500, 0.9% NaCl, dH_2_O) and mixed by turning. The red blood cells were allowed to fall to the bottom for 30 min at RT. The top layer was then moved into a clean tube and centrifuged for 10 minutes at 500 x g. The pellet was resuspended into PBS and pipetted on the top of Ficoll. The tubes were centrifuged 40 minutes without a brake at 400 x g, after which the top layers were carefully removed. The remaining red blood cells were lysed with 5 ml of dH_2_O for 28 seconds, after which equal volume of 2xPBS was added. The samples were centrifuged at 500 x g, 5 minutes and the lysis repeated 2-3 times. The cell pellets were stored at -80°C for protein extractions (described later).

Fibroblasts were established and cultured as previously described (20). 3 x 10^5^ cells were plated onto T25 flasks and cultured at 37°C and 5% CO_2_ in high-glucose DMEM with 100 U/ml penicillin, 100 μg/ml streptomycin, and 10% FBS (Corning) for 48 h and for additional 24 h with and without (40µM) Simvastatin (567021; Calbiochem). The cells were scraped to the lysis buffer to extract proteins (described later).

### 2.4 Annexin V/PI staining of PBMCs

CPT-isolated PBMCs from mutation carriers (III:1 and III:2) and sex-matched controls were thawed and plated 3 x 10^6^/ml in RPMI 1640 medium. The cells were allowed to rest overnight. The next day the cells were left unstimulated or stimulated with PMA/Cell Stimulation Cocktail (Invitrogen; 00-4975-93) for 2.5 hours. The cells were stained with FITC Annexin V/Dead Cell Apoptosis Kit (BD Biosciences; V13242) according to manufacturer’s instructions. The data was collected with BD LSRFortessa™ using BD FACSDiva software and analyzed with FlowJo™ 10 (BD Biosciences).

### 2.5 Western blot analysis

Proteins were extracted to Cell Lysis Buffer (9803S; Cell Signaling Technologies) with Halt™ Protease Inhibitor cocktail and phosphatase inhibitors (Thermo Fisher Scientific). The protein concentrations measured with DC protein assay (Bio-Rad Laboratories). Proteins (30 µg) in the sample loading buffer (8 M urea, 2% SDS, bromophenol blue) were separated by electrophoresis in reducing conditions on a 12% sodium dodecyl-sulfate polyacrylamide gel (SDS-PAGE) with PageRuler Plus Prestained protein ladder (Thermo Fisher Scientific; #26620) and transferred onto Immobilon^®^-FL PVDF membrane (Millipore). After blocking the membrane with Odyssey^®^ blocking buffer (LI-COR, Lincoln, NE, USA), 1 µg/ml anti-KLF2 antibody (Thermo Fisher Scientific; #PA5-40591) or 0.5 µg/ml mouse anti-GAPDH (Thermo Fisher Scientific; #437000) antibodies were allowed to bind o/n at 4°C or 2h at RT correspondingly. After incubation with 1:10 000 anti-mouse or anti-rabbit secondary antibodies (IRDye 680RD or 800CW) (LI-COR Biosciences) for one hour at room temperature (RT), the immune complexes were detected by Odyssey infrared scanner (LI-COR Biosciences). The band intensities were analyzed by Image Studio Lite Ver 5.2 from three individual analyses (LI-COR Biosciences).

### 2.6 KLF2 staining

CPT-isolated PBMCs from mutation carriers and sex-matched controls were thawed and plated 3 x 10^6^/ml in RPMI 1640 medium. The cells were allowed to rest overnight and thereafter live and dead cells were stained with LIVE/DEAD™ Fixable Near-IR Dead Cell Stain Kit (Invitrogen; L10119). The cells were washed twice in PBS (Sigma Aldrich; D1408) with 2% FBS and centrifuged at 550 x g for 2 minutes. The cells were stained for surface markers for 30 minutes at 4°C, washed, fixed and permeabilized with Transcription Factor Staining Buffer Set (Miltenyi Biotec, 130-122-981) for 30 minutes at 4C°. After permeabilization the cells were washed and stained for KLF2 for 35 minutes at 4C°. The antibodies against anti-human CD3 Alexa Fluor 488 (557694), anti-human CD4 PE Cy7 (557852) were purchased from BD Biosciences. The PE conjugated antibody against human KLF2 was purchased from Miltenyi Biotec (Miltenyi Biotec; 130-111-039). The data was collected with BD LSRFortessa™ using BD FACSDiva software and analyzed with FlowJo™ 10 (BD Biosciences).

### 2.7 Cellular distribution of KLF2 and KLF2 staining of isolated nuclei

KLF2 distribution in T cells was studied with flow cytometry. CPT-isolated PBMCs from healthy control were thawed and plated 3 x 10^6^/ml in RPMI 1640 medium. The cells were washed twice in PBS (Sigma Aldrich; D1408) with 2% FBS and centrifuged at 550 x g for 2 minutes. The cells were stained for surface markers for 30 minutes at 4°C, washed, fixed and permeabilized with Transcription Factor Staining Buffer Set (Miltenyi Biotec, 130-122-981) or with Cytofix/Cytoperm (BD Biosciences; 554714) for 30 or 20 minutes correspondingly at 4°C. The control samples without fixation and permeabilization were kept in PBS with 2% FBS. After fixation/permeabilization the cells were washed and stained for KLF2 and FOXP3 for 35 minutes at 4°C. FOXP3 was used as a control marker due to its known localization in the nucleus (not shown). The antibodies against anti-human CD3 Alexa Fluor 488 (557694), anti-human CD4 PE Cy7 (557852) were purchased from BD Biosciences. The APC conjugated antibody against human KLF2 was purchased from Miltenyi Biotec (Miltenyi Biotec; 130-111-040). The anti-human FOXP3 PE (12–4776–42) antibody was purchased from Invitrogen. The data was collected with BD LSRFortessa™ using BD FACSDiva software and analyzed with FlowJo™ 10 (BD Biosciences).

To isolate nuclei for KLF2 staining, 4-8 million PBMCs from mutation carrier and sex-matched controls isolated by Ficoll-Paque centrifugation were thawed at 37°C and washed with cold PBS. Nuclei were isolated with Minute ™ Detergent-free Single Nuclei Isolation kit (Invent Biotechnologies; NI-024) according to manufacturer’s instructions. Isolated nuclei were washed, fixed and permeabilized with the Transcription Factor Staining Buffer Set (Miltenyi Biotec, 130-122-981) for 10 minutes at 4°C. After permeabilization the nuclei were washed and stained for APC conjugated antibody against human KLF2 (Miltenyi Biotec; 130-111-040) along with isotype control (Miltenyi Biotec; 130-120-709) for 30 minutes at 4°C. The gating was subjected tightly only to the round and whole nuclei (based on FSC and SSC) that were approximately the same size as the lymphocyte population would have been. The nuclei were stained with DAPI (0.5 mg/ml) (Thermo Fisher Scientific; 62248). The data was collected with BD LSRFortessa™ using BD FACSDiva software and analyzed with FlowJo™ 10 (BD Biosciences).

### 2.8 Interactome analysis by mass spectrometry

KLF2 WT and KLF2 mutant (generated by insertion of additional nucleotide 951 to the WT sequence) were fused with MAC-Tag-N (21) (Addgene, Plasmid #108078) and MAC-Tag-N (Addgene, Plasmid # 108078) destination vector using Gateway cloning. Flp-In™ 293 T-REx cell line (Invitrogen, R78007) was used to generate isogenic and inducible stable cell lines from wild-type and mutant KLF2 as in Liu et al., 2020 (22). Each stable cell line was expanded to 80% confluence in 14 × 150 mm cell culture plates. Five plates were used for each replicate, in which 1 μg/ml tetracyclin and 50 μM biotin was added for 24 h before harvesting. Cells from each replicate (n=3) were pelleted and snap-frozen, and stored at −80 °C until analysis.

For BioID analysis the cell pellets were lysed, affinity purified with Strep-Tactin beads (IBA, GmbH), and digested to tryptic peptides as described in Liu et al., 2020 (22). After quenching with 10% TFA, the samples were desalted with C18 MicroSpin columns according to the manufacturer’s instructions (Harvard Apparatus, USA). The eluted peptide sample was dried in a vacuum centrifuge and reconstituted to a final volume of 30 μl in buffer A (0.1% trifluoroacetic acid and 1% acetonitrile in HPLC water). Samples were further diluted 1 + 19 µl with HPLC water containing 0.1% formic acid. The manufacturer’s instructions were followed to load into Evotips (Evosep).

The desalted samples were analyzed using the Evosep One liquid chromatography system coupled to a hybrid trapped ion mobility quadrupole TOF mass spectrometer (Bruker timsTOF Pro) *via* a CaptiveSpray nano-electrospray ion source. An 8 cm × 150 µm column with 1.5 µm C18 beads (EV1109, Evosep) was used for peptide separation with the 60 samples per day methods (21 min gradient time). Mobile phases A and B were 0.1% formic acid in water and 0.1% formic acid in acetonitrile, respectively. The MS analysis was performed in the positive-ion mode using data-dependent acquisition (DDA) in PASEF (Meier, Brunner et al., 2018) mode with DDA-PASEF-short_gradient_0.5s-cycletime -method.

Raw data (.d) were processed with FragPipe v17.1 utilizing MSFragger (23) against reviewed human entries of the UniProtKB database (downloaded 8.3.2022). Carbamidomethylation of cysteine residues was used as static modification. Aminoterminal acetylation and oxidation of methionine were used as the dynamic modification. Biotinylation of lysine and N-termini were set as variable modifications. Trypsin was selected as an enzyme, and a maximum of two missed cleavages were allowed. Both instrument and label-free quantification parameters were left to default settings. Final results from these steps are Spectral Counts (SC) values from peptides with FDR < 0.01 from Philosopher.

Significance Analysis of INTeractome (SAINT) -express version 3.6.3 (24) and Contaminant Repository for Affinity Purification (CRAPome, Mellacheruvu et al., 2013) were used to discover statistically significant interactions from the data. Results represent proteins with a BFDR < 0.05, and in less than 20% of Crapome database experiments except in cases where AvgSpec is three times higher than AvgSpec in Crapome experiments. Data visualization was performed with Prohits-viz.org and proteomics.fi webtools.

### 2.9 Staining of S1PR1 and CCR7

PBMCs isolated by Ficoll-Paque gradient centrifugation from mutation carrier and sex-matched controls were thawed and plated 3 x 10^6^/ml in RPMI 1640 medium. The cells were allowed to rest overnight and thereafter live and dead cells were stained with LIVE/DEAD™ Fixable Near-IR Dead Cell Stain Kit (Invitrogen; L10119). The cells were washed twice in PBS (Sigma Aldrich; D1408) with 2% FBS and centrifuged at 550 x g for 2 minutes. The cells were stained for surface markers for 30 minutes at 4°C, washed, fixed with 4% formaldehyde (Thermo Fisher Scientific; 28908) in PBS for 10 minutes at room temperature. The antibodies against anti-human CD3 Alexa Fluor 488 (557694), anti-human CD4 PE Cy7 (557852), anti-human CD19 PE CF594 (562294), anti-human CCR7 PE (566741) were purchased from BD Biosciences. The eFluor^®^ 660 conjugated antibody against human CD363 (S1PR1) was purchased from Invitrogen (Invitrogen; 50-3639-42). The data was collected with BD LSRFortessa™ using BD FACSDiva software and analyzed with FlowJo™ 10 (BD Biosciences).

### 2.10 B and T cell immunophenotyping and vaccine response

B and T lymphocyte immunophenotyping was completed from fresh heparin-blood samples. Four or 10-color flow cytometry panel with monoclonal antibodies (mAb) against the surface antigens IgM, IgD, CD3, CD4, CD8, CD16҄56, CD19, CD21, CD27, CD33, CD34, CD38, CD45, CD56, CD57, CD133, HLA-DR, CD62L, CD45RA and CD45RO (BD Biosciences). T cell immunophenotyping was studied with the antibody panel including anti-CD45, -CD3, -CD4, -CD8, -CD45RA, and -CCR7 (R&D Systems). B and T cell phenotyping was completed as previously described and B and T cell subpopulations were analyzed according to published protocols (25–27). The vaccine response to pneumococcal polysaccharide vaccine (Pneumovax^®^) was performed as described (28).

### 2.11 Whole blood staining of B1 B cells

Blood was collected from mutation carrier and sex-matched controls in lithium-heparin tubes. For analysis of surface markers, 300µl blood was stained for 30 minutes at room temperature: Red blood cells were lysed with BD FACS™ Lysing Solution (BD Bioscience; 349202) for 10 minutes at room temperature, washed, fixed with 4% formaldehyde in PBS for 10 minutes at room temperature. The antibodies against anti-human CD3 PE CF594 (562406), against anti-human CD4 PE CF594 (566914), against anti-human CD7 PE CF594 (562541), anti-human CD38 PE Cy7 (335808), anti-human CD27 APC (558664), anti-human CD20 BV480 (566132), anti-human CD43 BB515 (Biosciences; 564542) and anti-human CD19 PE (555413) were purchased from BD Biosciences. The data was collected with BD LSRFortessa™ using BD FACSDiva software and analyzed with FlowJo™ 10 (BD Biosciences).

### 2.12 T helper cell differentiation

IL-17 positive Th17 cells were analyzed with accepted methods. Briefly, fresh PBMCs were stimulated for 16 hours with anti-CD3/anti-CD28 beads (Life Technologies) in the presence of Brefeldin A (Sigma-Aldrich). Thereafter, the cells were fixed, permeabilized and stained with anti-CD4, -CD69-APC, and -IL-17A (BD Biosciences), analyzed with BD LSRFortessa flow cytometer (BD Biosciences) and using FlowJo software (BD Biosciences). Th17 CD4+ memory cells were detected from whole blood by a four-color flow cytometry panel with monoclonal antibodies (mAbs) [CD45RA-FITC, CD4-PerCP, CXCR3-APC and CCR6-BV421 (BioLegend)] against surface antigens.

### 2.13 Staining of regulatory T cells

PBMCs isolated by Ficoll-Paque gradient centrifugation from mutation carrier and sex-matched controls were thawed and plated 3 x 10^6^/ml in RPMI 1640 medium. The cells were allowed to rest for 2 hours and thereafter live and dead cells were stained with LIVE/DEAD™ Fixable Near-IR Dead Cell Stain Kit (Invitrogen; L10119). The cells were washed twice in PBS (Sigma Aldrich; D1408) with 2% FBS and centrifuged at 550 x g for 2 minutes. The cells were stained for surface markers in Brilliant Stain Buffer (BD Biosciences; 563794) for 30 minutes at 4°C, washed, fixed and permeabilized with Transcription Factor Staining Buffer Set (Miltenyi Biotec, 130-122-981) for 30 minutes at 4°C. After permeabilization the cells were washed and stained for FOXP3 for 35 minutes at 4°C. The antibodies against anti-human CD4 BV510 (562970), anti-human CD127 PE Cy7 (560822), anti-human CD45RA APC (561210), anti-human CD25 BV421 (BD Biosciences; 564033), anti-human CCR7 PE (566741), anti-human CCR6 PE (551773) and anti-human CXCR3 PE (550633) were purchased from BD Biosciences. The Alexa Fluor 488 conjugated antibody against human FOXP3 was purchased from Biolegend (Biolegend; 320112) The data was collected with BD LSRFortessa™ using BD FACSDiva software and analyzed with FlowJo™ 10 (BD Biosciences).

### 2.14 NanoString analysis

NanoString mRNA counting was performed as previously described (29). CPT-isolated PBMCs from mutation carriers and age/sex-matched controls were snap frozen in liquid nitrogen and stored at -80°C. The total RNA from 2.5 x 10^6^ cells was isolated using RNeasy Minikit (74106; Qiagen) according to the manufacturer’s instructions. 100 ng of RNA was used for NanoString (NanoString Technologies) gene expression analysis. Each sample was analyzed once. Custom gene panel contains in total 50 genes including 45 IFN-regulated, inflammasome-related, as well as JAK/STAT and NF-κB signaling pathway genes, and five housekeeping genes. As housekeeping genes, we used elongation factor 1-gamma (EF1G), glyceraldehyde-3-phosphate dehydrogenase (GAPDH), hypoxanthine-guanine phosphoribosyltransferase (HPRT1), ornithine decarboxylase antizyme 1 (OAZ1), and tubulin beta class 1 (TUBB). For overnight hybridization at 65°C, RNAs were mixed with 5′ reporter probes (tagged with fluorescent barcodes of targeted genes) and 3′ biotinylated capture probes. Reactions were purified and immobilized on the sample cartridge surface by utilizing the Prep Station (NanoString Technologies), and the cartridge was scanned in triplicate using an nCounter Digital Analyzer (NanoString Technologies). Gene expression data was analyzed with the nSolver™ 4.0 analysis software (NanoString Technologies) performing two-step normalization using default settings as recommended by the manufacturer. The normalized values were utilized to calculate fold change for each individual gene between patient and matched control.

### 2.15 RelA/p65 phosphorylation

CPT-isolated PBMCs from mutation carriers and sex-matched controls were thawed and plated 2.5 x 10^6^/ml in RPMI 1640 medium. The cells were allowed to rest for a minimum of 2 hours and thereafter the cells were washed once in PBS with 2% FBS, centrifuged at 550 x g for 2 minutes and stained with LIVE/DEAD™ Fixable Violet Dead Cell Stain Kit (Invitrogen; L34955). The cells were left unstimulated or stimulated with PMA/Cell Stimulation Cocktail (Invitrogen; 00-4975-93) or lipopolysaccharide (LPS) solution (Invitrogen; 00-4976-93) for 15 minutes, immediately fixed 10 minutes in 4% formaldehyde (Thermo Fisher Scientific; 28908) in PBS at RT and washed twice as above. Next, the cells were permeabilized with ice cold Perm Buffer III (BD Biosciences; 558050) for 30 minutes on ice, washed and finally stained for 35 minutes at RT: anti-human CD3 FITC (Thermo Fisher Scientific; 11-0036-42), anti-human CD4 PE Cy7 (BD Biosciences;557852) and anti-human NF-kB p65 Phosphorylation pS529 Alexa Fluor 647 (BD Biosciences; 558422). The data was measured with BD LSRFortessa™ using BD FACSDiva software and analyzed with FlowJo ™ 10 software (BD Biosciences).

### 2.16 Inflammasome activity assay

The activation of inflammasomes was examined as described (30). Shortly, 0.75 x 10^6^ cells in 0.5 ml were seeded onto a 24-well plate in RPMI 1640 (Sigma Aldrich; R0883) supplemented as above. The next day, 1µg/ml LPS (Sigma Aldrich; L3012) was applied to the cells for 6 hours followed by additional 45-minute incubation with 5mM ATP (Sigma Aldrich; A6419). The cells were centrifuged (300 x g, 5 min) and the media collected and stored at -80°C for future analyses. Il-1β was measured from media using a commercial enzyme-linked immunosorbent assay (ELISA) (R&D Systems; DY201) according to manufacturer’s instructions.

## 3 Results

### 3.1 Genetic analysis

**Figure 2:**
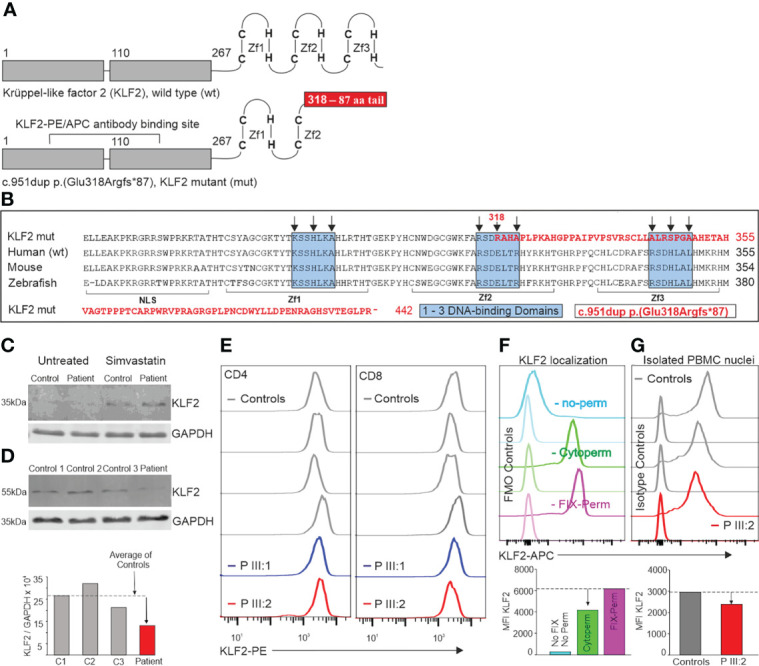
**(A)** KLF2 protein structure. The conserved C2H2-type zinc finger structure at the C-terminus consists of three loops that are crucial for DNA-binding. The KLF2 c.951dup p.(Glu318Argfs*87) mutation is located in the middle of the second zinc finger loop. The duplication of Glu318 causes a shift in the reading frame which leads to a stop codon 87 amino acids later. **(B)** Comparison of the sequence coding the human mutant to human, mouse, and zebrafish with KLF2 zinc fingers. The mutated part of the KLF2 sequence [c.951dup p.(Glu318Argfs*87)] is typed with red color. The sequence shown begins with the nuclear localization signal (NLS: amino acids (aa) 254 - 274), followed by the first zinc-finger (Zf1: aa277 - 297), the second zinc-finger (Zf2: aa304 - 327) and the third zinc finger (Zf3: aa334 - 254). Within the zinc finger sequences, there are three conserved DNA-binding domains (marked with blue boxes). The black arrows point at important amino acids that bind directly to DNA. The KLF2-PE/APC antibody binding sequences **(A)** are intact both in the wildtype and the variant alleles. KLF2 was detected by western blot in patient (III:2) skin fibroblasts **(C)** and neutrophils **(D)** as 37 kDa and 60 kDa molecular weight forms correspondingly. 30µg of protein was separated on a 12% gel and transferred into PVDF membrane. The KLF2 and GAPDH antibody binding was detected by anti-mouse or anti-rabbit secondary antibodies (IRDye 680RD or 800CW). The membranes were imaged by Odyssey infrared scanner and band intensities quantified from three different sample sets. **(E)** KLF2 expression in CD4^+^ and CD8^+^ lymphocytes. The KLF2 expression of isolated PBMCs from mutation carriers (III:1 and III:2) and sex-matched controls was determined by flow cytometry. **(F)** Localization of KLF2 protein was studied in control PBMCs with flow cytometry using three different permeabilization strategies: first without any permeabilization/fixation (used when staining cell membrane targets), second with Cytoperm buffer (used when staining cytoplasmic targets) and finally, a Fixation/permeabilization buffer (used when staining nuclear transcription factors). Quantitation of MFI values is shown in bar chart below. **(G)** Nuclei from patient and control PBMCs were isolated, fixed, permeabilized and stained with KLF-APC and isotype control antibodies. Quantitation of MFI values is shown in bar chart below.

The unique clinical and immunological manifestations (**Figure 1**) combined with lymphocyte abnormalities of the index patient IV:2 prompted us to perform whole-exome sequencing (WES). A novel c.951dup, p.(Glu318Argfs*87) variant in exon 3 of the krüppel-like factor 2 (KLF2) gene (NM_016270.3, chr19(GRCh37):g.16437725dup) was identified in the index patient and her mother. The same variant was also found in the several other family members (**Figure 2A**). This novel KLF2 variant causes a shift in the reading frame starting at codon 318 and the new reading frame ends in a stop codon 86 positions downstream (**Figure 2**). The variant is localized within the highly conserved KLF2 zinc finger domain that consists of three loops; the mutation is predicted to affect the structure of the second and third zinc finger loops (**Figures 2B, C**) (31). As demonstrated in **Figure 2**, the mutation deletes the conserved amino acids in the second and third zinc-finger structures. These sequences are required for nuclear entry and DNA binding (32–34). The KLF2 p.(Glu318Argfs*87) variant is rare, and it is absent in FinnGen, ClinVar, CentoMD and gnomAD databases. No other significant genetic variants were identified. Despite remarkable abnormalities in patients’ lymphocytes, no differences in the number of dead cells (observed by LIVE/DEAD Stain Kit) or apoptosis (examined by Annexin V/Dead Cell Apoptosis Kit) was observed during the immunological characterizations *in vitro* (data not shown).

### 3.2 KLF2 protein expression

To examine whether the mutant KLF2 protein is detectable in patient’s cells, we first examined proteins extracted from skin fibroblasts. By western blotting, the wild type KLF2 protein with a molecular weight of ~37kDa (as predicted based on its expected size) was detected, in both control and patient fibroblasts, but only after simvastatin treatment (**Figure 2C**, **Supplementary material**). Simvastatin has been previously shown to increase the expression of KLF2 (35). Both control and patient cells also showed higher molecular weight immune complexes, with approximate molecular size of ~130kDa and higher, which may be due to post- translational modifications, such as ubiquitination (36). However, no KLF2 variants, exclusively in patient cells were detected, which could correspond to the larger mutant form.

We next examined whether the mutant form of KLF2 could be detected in other cell types from the patient. KLF2 is expressed in neutrophils (37) and based on our data, KLF2 mutation does not affect the number of neutrophils. Thereby, we were able to isolate an adequate number of these immune cells from the patient to perform western blot analysis. Interestingly, in neutrophils KLF2 was observed as a single band with molecular weight of approximately 60kDa in both control and patient cells. KLF2 might be post-translationally modified, e.g. ubiquitinated (36). Alternatively, KLF2 may form highly stable complexes with other proteins. Again, no mutant protein was detected. However, the patient cells expressed only half of the levels of wild type KLF2 compared to the controls (**Figure 2D**, **Supplementary material**). As KLF2 variant positive patients show no difference in neutrophil counts, it would seem likely that expression of KLF2 is not crucial for the development or survival of these cells.

The KLF2 expression in PBMCs was analyzed using a commercial KLF2 antibody recognizing parts of transactivation and transrepression KLF2 domains (amino acids 71-168) which are intact both in the wild-type and the mutated p.(Glu318Argfs*87*) allele and hence, the antibody does not differentiate the wild-type or the mutated gene products from each other. Flow cytometric analysis showed that KLF2 expression in patients’ T cells is comparable to controls or only slightly reduced (**Figure 2E**). The perhaps unlikely explanation could be that both wild-type and mutant alleles are equally present in these cells and that the protein of the p.(Glu318Argfs*87*) mutant allele is not subject to post-translational degradation or downregulation. However, the lymphopenic state of the patients would more likely suggest that the KLF2 detected by flow cytometry is mostly of the wild type and those cells expressing the mutant variant would fail to mature or survive, thereby giving a reasonable explanation to the lower lymphocyte numbers in the patients.

### 3.3 Nuclear entry of KLF2

Flow cytometric analysis showed that approximately 66% of KLF2 was localized in the cytoplasm in unstimulated T cells. Approximately 34% higher KLF2 MFI value was observed when the fixation/permeabilization allowed the antibody to reach the nucleus, compared with the conditions allowing the cytoplasmic staining only (**Figure 2F**). Since FOXP3 is known to localize in the nucleus exclusively, it was used as a control marker to demonstrate that the permeabilization conditions worked correctly. As expected, a normal FOXP3^+^ population among the CD4^+^ T cells was observed only when using the Fix-Perm buffer that permeabilizes both plasma membrane and the nuclear membrane (data not shown). A 20% decrease in the KLF2 MFI value in the patient III:2 nuclei was observed compared to controls (**Figure 2G**). This change, although not substantial, suggests that the KLF2 variant reported here may affect the entrance of mutant KLF2 protein to the nucleus.

### 3.4 Interactome results

**Figure 3:**
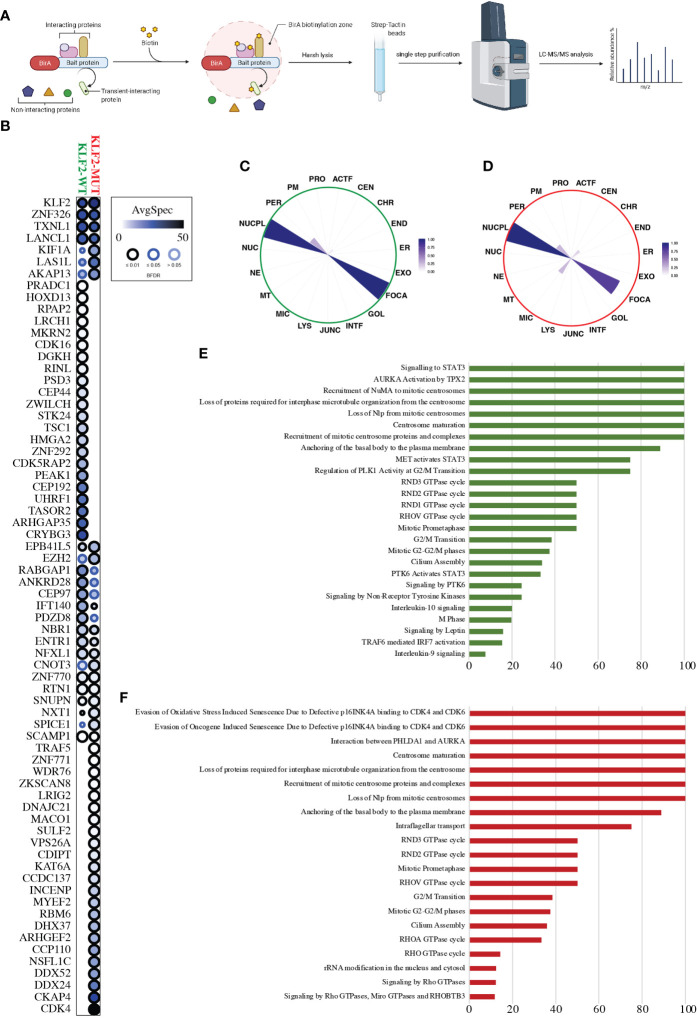
KLF2 interactome analysis. **(A)** Schematic workflow overview of the MAC-tagged based BioID purification coupled with mass spectrometry. **(B)** Dot-plot visualization (BFDR ≤ 0.05) of the KLF2-WT and KLF2-MUT interactors (prohits-viz.org). Each node corresponds to the abundance of the average spectral count for each prey. **(C, D)** The polar plot shows the molecular level localization of KLF2-WT and KLF2-MUT obtained by MS-microscopy respectively (proteomics.fi). ACTF, Actin filament; JUNC, Cell junction; CEN, Centrosome; CHR, Chromatin; ER, Endoplasmic reticulum; END, Endosome; EXO, Exosome; FOCA, Focal adhesion; GOL, Golgi; INTF, Intermediate filament; LYS, Lysosome; MIC, Microtubule; MT, Mitochondrion; NE, Nuclear envelope; NUC, Nucleolus; NUCPL, Nucleoplasm; PER, Peroxisome; PM, Plasmamembrane; PRO, Proteasome. **(E, F)** Significantly enriched (q < 0.05, calculated with Fisher exact test and Benjamini–Hochberg multiple-testing correction) Reactome pathway annotations from the KLF2-WT and KLF2-MUT interactors, respectively. Y-axis corresponds to the percentage of the interactors found in the given pathway.

We analyzed close-proximity protein-protein interactions (PPIs) of wild-type and mutant KLF2 using mass spectrometry (**Figure 3A**). BioID analysis revealed 46 high-confidence PPIs for the wild-type KLF2, of which 24 were detected in the mutant (**Figure 3B**). As the total number of detected high-confidence PPIs in the mutant KLF2 was 47, it had gained novel interactions with 23 proteins (**Figure 3B**). Among the gained interactors was CDK4, a protein involved in cell cycle progression from G1 to S phase. MS microscopy showed a similar subcellular localization for wild-type and mutant KLF2 (**Figures 3C, D**). We then analyzed the pathways regulated by the KLF2 interacting proteins. For the wild-type KLF2 several pathways related to immune regulation were identified. These included ‘signaling to STAT3’ (100%), ‘AURKA Activation by TPX2’ (100%), ‘MET activates STAT3’ (75%), ‘PTK6 activates STAT3’ (33%), ‘Signaling by PTK6’ (24.5%), ‘Signaling by Non-Receptor Tyrosine Kinases’ (24.5%), ‘Interleukin-10 signaling’ (20%), ‘TRAF6 mediated IRF7 activation’ (15.3%), and ‘Interleukin-9 signaling’ (7.7%). In the KLF2 mutant these Reactome pathways were absent (**Figures 3E, F**). Interestingly, Reactome pathways ‘Evasion of Oxidative Stress Induced Senescence Due to Defective p16INK4A binding to CDK4 and CDK6’ (100%), ‘Evasion of Oncogene Induced Senescence Due to Defective p16INK4A binding to CDK4 and CDK6’ (100%), ‘Interaction between PHLDA1 and AURKA’ (100%), ‘RHOA GTPase cycle’ (33.3%), and ‘RHO GTPase cycle’ (14.2%) were only detected in the mutant KLF2.

### 3.5 KLF2 p.(Glu318Argfs*87) variant is associated with B cell abnormalities

KLF2 deficiency affects the B cell maturation and migration in experimental models (38). The KLF2 p.(Glu318Argfs*87) variant positive family members were lymphopenic and their peripheral blood CD19^+^ B cells and B cell subsets were also low (**Table 1**). In particular, the CD27^+^IgD^-^IgM^-^ switched memory B cells were low. Percentages of CD38^low^CD21^low^ activated B cells in turn were elevated, consistent with previous observations in autoimmune conditions (39, 40). The immunoglobulin concentrations and vaccine responses were mostly normal although variation in IgG1 concentrations were seen (**Table 1**). The index case (IV:2) affected by juvenile idiopathic arthritis displayed a low serum IgA (0.35g/L), IgG (3.9g/L) and IgG subclasses already before immunomodulatory treatments. The laboratory findings of the index patient IV:2 fulfills the European Society for Immunodeficiency (ESID) criteria of common variable immunodeficiency (CVID) (41). The reduced polysaccharide vaccine response of the IV:2 (**Table 1**), however, was analyzed during methotrexate treatment (42). Only this index patient IV:2 receives IgG substitution. The other KLF2 variant positive family members have not presented with obvious primary infection susceptibility typical for B cell deficiency. However, they have suffered from infections followed by their immunomodulatory treatments (patients II:1, II:2). A whole blood staining *(ex vivo)* of B1 B cells (CD20^+^CD19^+^CD27^+^CD43^+^CD38^low/int^) of patient III:2 and matched controls showed that the patient had normal percentage (3.9% vs controls 2.8-8.5%) and even normal number of B1 B cells compared with controls (data not shown).

**Table 1:** Immunophenotype of patients with KLF2 [c.951dup p.(Glu318Argfs*87)] variant.

Patient	II:1	II:2	III:1	III:2	IV:1		IV:2	
Age at sampling	44	73	37	48	20		13	
Gender	M	M	M	F	M		F	
p.(Glu318Argfs*87*)	+/-	+/-	+/-	+/-	+/-		+/-	
						**Range (adult)**		Range (child)**
**Leukocytes**	18.0*	4.5	5.7	3.3	2.7	3.4-8.2×10^9^/L	2.7↓	4.5-13.5×10^9^/L
Lymphocytes	12.75*	0.5↓	0.8↓	0.4↓	0.3↓	1.2-3.5×10^9^/L	1.7	1.4-6.8×10^9^/L
Monocytes	0.4*	0.4	0.7	0.3	0.4	0.2-0.8×10^9^/L	0.3	<0.18×10^9^/L
Neutrophils	3.76*	3.6	4.1	2.5	2.0	1.6-6.3×10^9^/L	1.6	1.6-9.5×10^9^/L
Basophils	0.05*	0.02	0.03	0.02	0.01	<0.09×10^9^/L	0.02	<0.05×10^9^/L
Platelets	286*	147	233	189	154	150-360×10^9^/L	318	200-450×10^9^/L
NK cells	–	31	111	53	78	84-724×10^6^/L	99	80-1770×10^6^/L
**CD3^+^**	–	442↓	806	214↓	214↓	742-2750×10^6^/L	1186	784-5848×10^9^/L
TCRαβ^+^	–	97.2	99.2	94.5	97.7	88.1-97.8%	98.0	na
TCRγδ^+^	–	2.8	0.8	5.5	2.3	1.9-11.7%	2.0	na
CD4^-^CD8^-^TCRαβ^+^	–	0.1	0.2	0.6	0.7		1.5	na
RTE CD45RA^+^CD62L^+^CD31^+^	–	8↓ (1.9)	155 (19.2)	18↓ (8.6)	31↓ (14.4)	107-1053×10^6^/L 14.4-38.3%	415 (35.0)	na
CD62L^+^	–	184↓ (41.6)	636 (78.9)	143↓ (66.8)	174↓ (81.2)	441-1840×10^6^/L 59.4-66.9%	950 (80.1)	na
**CD4/CD8**	–	1.3	2.7	1.8	2.1	0.6-2.8	1.3	
**CD3^+^CD4^+^**	–	249↓	607	134↓	145↓	404-1612×10^6^/L	630	462-3940×10^9^/L
TCM CCR7^+^CD45RA^-^	–	107 (42.8)	357 (58.9)	90 (67.3)	87 (60.3)	34-529×10^6^/L (8.4-32.8%)	265 (42.0)	na
Naive CCR7^+^CD45RA^+^	–	17↓ (6.9)↓	196 (32.3)	15↓ (11.3)↓	28↓ (19.6)↓	83-883×10^6^/L (20.5-54.8%)	281 (44.6)	na
TEM CCR7^-^CD45RA^-^	–	51↓ (20.3)	52↓ (8.5)	29↓ (21.5)	29↓ (20.1)	80-847×10^6^/L (19.9-52.4%)	83 (13.2)	na
TEMRA CCR7^-^CD45RA^+^	–	75 (30.0)	2↓ (0.3↓)	0↓ (<0.3↓)	0↓ (<0.2↓)	6-274×10^6^/L (1.4-17.0%)	1↓ (0.2↓)	na
CD62L^+^	–	123↓ (49.4)	518 (85.4)	103↓ (77.3)	119↓ (81.8)	284-1415×10^6^/L (70.4-87.8%)	488 (77.4)	na
**CD3+CD8+**	–	187↓	222	75↓	68↓	220-1129×10^6^/L	503	
Naive CCR7^+^CD45RA^+^	–	12↓ (6.4)	111 (49.8)	25↓ (33.0)	47 (69.0)	41-802×10^6^/L 18.8-71.0%	423 (84.1)	na
TCM CCR7^+^CD45RA^-^	–	10 (5.5)	21 (9.4)	10 (12.9)	5 (7.8)	3-82×10^6^/L (1.2-7.3%)	24 (4.8)	na
TEM CCR7^-^CD45RA^-^	–	22↓ (11.5)	39 (17.6)	21↓ (28.0)	12↓ (17.2)	32-711×10^6^/L (14.6-63.0%)	43 (8.6)	na
TEMRA CCR7^-^CD45RA^+^	–	143 (76.6)	52 (23.2)	20 (26.1)	4↓ (6.0)	10-380×10^6^/L (4.5-33.7%)	13 (2.6)	na
CD62L^+^	–	78↓ (41.7)	138 (62.0)	40↓ (53.3)	58↓ (84.7)	80-623×10^6^/L (36.3-55.2%)	442 (87.9)	na
**CD19^+^**	–	63↓	96	56↓	78↓	80-616×10^6^/L	431	70-1496×10^6^/L
Transitional CD38^hi^IgM^hi^	–	3 (5.0)	2 (2.3)	2 (4.3)	1 (1.5)	1-22×10^6^/L (0.6-3.5%)	19 (4.3)	na
Naive CD27^-^IgD^+^ IgM^+^	–	39 (62.2)	60 (62.4)	37 (66.4)	16↓ (19.9)↓	35-508×10^6^/L (43.2-82.4%)	246 (57.0)	na
Memory CD27^+^	–	37.5	37.1	33.0	79.6	na	39.2	na
Marginal zone CD27^+^IgD^+^IgM^+^	–	17 (27.4)	25 (25.9)	16 (28.4)	53 (67.8)	6-190×10^6^/L (7.2-30.8%)	139 (32.2)	na
Switched memory CD27^+^IgD^-^IgM^-^	–	2↓ (2.4)↓	8 (7.9)	0↓ (<0.7)↓	4↓ (5.5)↓	5-180×10^6^/L (6.5-29.2%)	12 2.8↓	na
Plasmablasts CD38^++^IgM^-^	–	<0.2↓	<0.3↓	<0.2↓	<0.3↓	0.4-3.6%	0.5	na
Activated CD38^low^CD21^low^	–	14.4↑	5.9	10.3↑	30.1↑	0.8-7.7%	19.8↑	na
**Immunoglobulins**								
IgG	13.5	10.4	9.8	8.5	8.2	6.77-15 g/L	3.9↓	6.0-14.4g/L
IgA	1.97	2.01	2.78	0.97	1.62	0.88-4.84 g/L	0.35↓	0.4-2.3g/L
IgM	1.44	0.96	0.74	0.86	0.65	0.36-2.59 g/L	0.52	0.4-2.1g/L
IgE	12	74	222	10	23	<110 U/L	<2	<130
IgG1	–	–	–	3.7↓	3.98↓	4.9-11.4 g/L	3.00↓	3.77-11.31g/L
IgG2	–	–	–	>6.2↑	3.52	1.5-6.4 g/L	1.06	0.68-3.88g/L
IgG3	–	–	–	0.35	0.35	0.2-1.1 g/L	0.14↓	0.16-0.89g/L
IgG4	–	–	–	<0.11	0.04	0.11-1.4 g/L	0.02	0.012-1.7g/L
**Vaccine response**	–	10/10	–	7/7	10/10		2/10↓***	

**Reference values B and T cell immunophenotyping for children are not available or based on estimates.

***The patient IV:2 received methotrexate treatment during the pneumococcus polysaccharide (Pneumovax^®^) response testing. All other laboratory values were analyzed before immunosuppressive treatments.

*The blood cell counts were analyzed prior to CLL diagnosis.

↑, increased; ↓, decreased; +/-, heterozygous for KLF2 genetic variant; na, not available.

### 3.6 Low peripheral blood T cells in the KLF2 p.(Glu318Argfs*87) variant patients

KLF2 deficiency causes loss of peripheral T cells in experimental models (8, 9). Consistent with the previous results, most of the KLF2 variant positive family members had low CD3^+^, CD3^+^CD4^+^ and CD3^+^CD8^+^ T cell counts (**Table 1**). Recent thymic emigrant (RTE) CD45RA^+^CD62L^+^CD31^+^ and naive CD3^+^CD4^+^CCR7^+^CD45RA^+^ T cells were reduced. The KLF2 variant positive family members III:2 and IV:1 had normal numbers of IL-17 positive Th17 cells compared to healthy controls.

### 3.7 CD62L, CCR7 and S1PR1 expression in KLF2 p.(Glu318Argfs*87) variant

**Figure 4:**
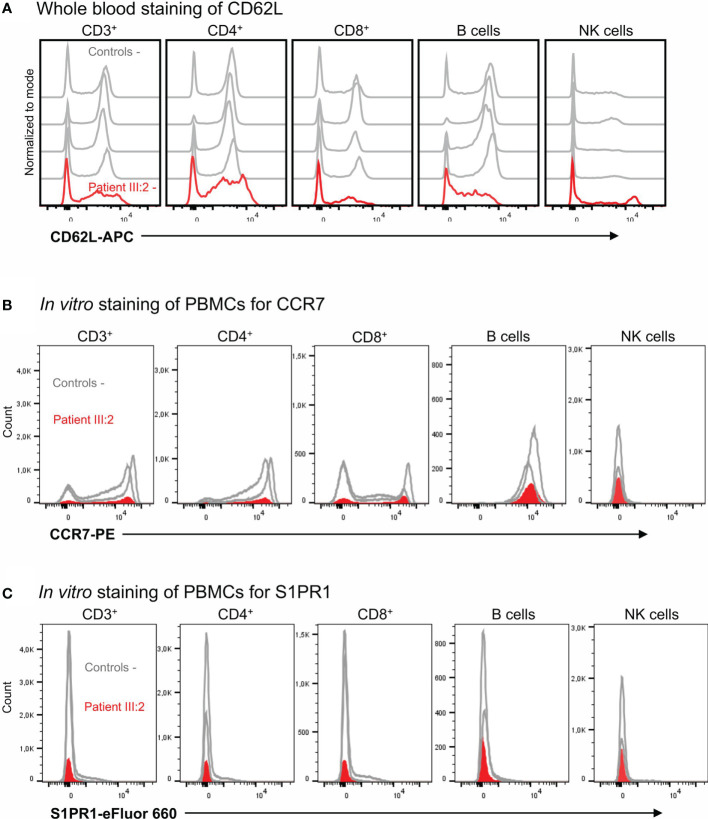
**(A)** Whole blood staining of CD62L. The CD62L expression in T cells, B cells and NK cells with flow cytometry. **(B)** *In vitro* staining of PBMCs for CCR7 expression. The CCR7 expression in T cells, B cells and NK cells with flow cytometry. **(C)** *In vitro* staining of PBMCs for S1PR1. The S1PR1 expression was studied in T cells, B cells and NK cells with flow cytometry.

Peripheral T cell lymphopenia in KLF2 deficiency is at least partly thought to be caused by low amounts of CD62L, an important lymphoid homing receptor. In these family members, the CD62L mean fluorescence index (MFI) and the numbers of CD62L positive cells was reduced both in CD3^+^CD4^+^ and CD3^+^CD8^+^ lymphocytes (**Figure 4A**) although the percentages of CD62L^+^ cells among the CD3^+^CD4^+^ and CD3^+^CD8^+^ lymphocytes were not severely affected (**Table 1**). The number of CCR7 and S1PR1 positive cells was very low (**Figure 4B, C**). Among the CCR7 and S1PR1 positive cells, the MFI values and the percentages of positive cells were at comparable levels between the patient and control.

### 3.8 Regulatory T cells in KLF2 p.(Glu318Argfs*87) variant patients

**Figure 5:**
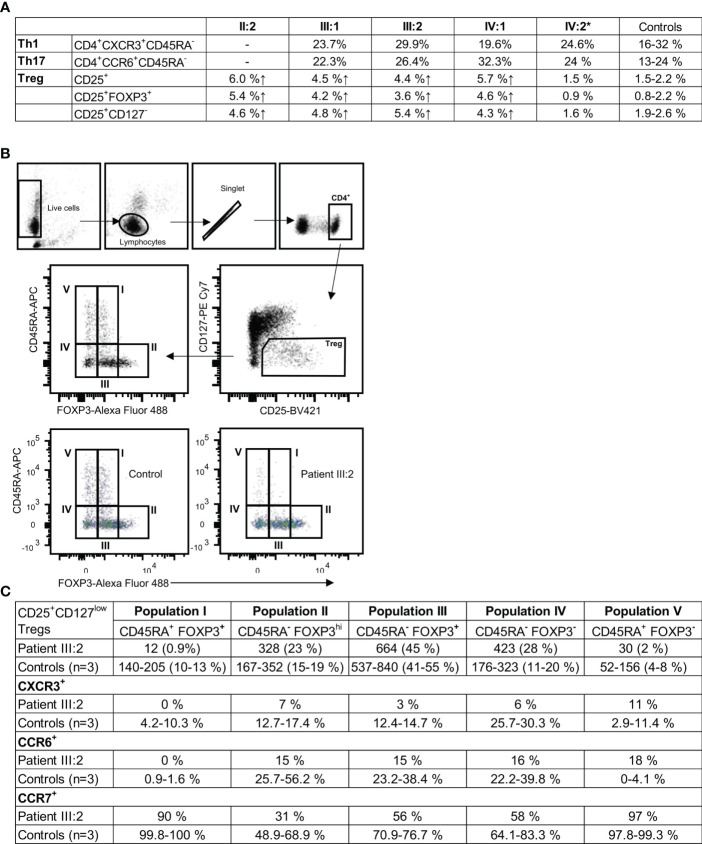
**(A)** CD3^+^CD4^+^ T cell subgroups in KLF2 c.951dup p.(Glu318Argfs*87) variant positive family members compared to healthy controls. Mutation carriers´ and controls´ unstimulated PBMCs were stained for regulatory T helper cell markers CD4, CD25, CD127 and FOXP3 and analyzed by flow cytometry. Th1 and Th 17 subpopulations were stained *ex vivo* (whole blood) with CD4, CXCR3, CCR6, CD45RA and CD10 antibodies and analyzed by flow cytometry. **(B)** Representative results of flow cytometry analysis of CD25^+^CD127^low^FOXP3^+^ regulatory T cells (Treg). The gating strategy is presented along with the data. Regulatory T cells of patient (III:2) and sex-matched controls´ were studied for the expression of CD45RA, CCR6, CCR7 and CXCR3 with flow cytometry. **(C)** The Treg populations I-V were studied for the expression of CCR6, CCR7 and CXCR3. Representative results of patient and control Treg populations are shown. The regulatory T cell populations I, II, III, IV and V are presented as the number of cells and percentages. The percentage of CXCR3, CCR6 and CCR7 positive cells.

In KLF2 variant family members without immunomodulatory medication, the percentages of CD3^+^CD4^+^CD25^+^ (4.4-6.0% patients; 1.5-2.3% controls), CD3^+^CD4^+^CD25^+^FOXP3^+^ (3.6-5.4% patients; 0.7-1.9% controls), and CD3^+^CD4^+^CD25^+^CD127^-^ (4.3-5.4% patients: 2.0-2.6% controls) Treg cells were elevated (**Figure 5**). In peripheral blood, the MFI for FOXP3 in the KLF2 variant positive CD3^+^CD4^+^CD25^+^FOXP3^+^ Treg cells was comparable to the controls. The CD25^+^CD127^low^ Treg were subdivided into five populations: population I: naïve/resting CD45RA^+^FOXP3^+^ Tregs; population II: activated CD45RA^-^FOXP3^hi^ Tregs with high FOXP3 expression; population III: CD45RA^-^FOXP3^+^; population IV: CD45RA^-^FOXP3^-^; and population V: CD45RA^+^FOXP3^-^ (**Figure 5B**). Populations IV and V are not true regulatory T cells since they lack the expression of FOXP3, whereas the population III consists of effector CD4^+^ T cells with transient CD25 and FOXP3 expression (43).

The naïve Treg population (population I) is nearly absent (patient 12 cells; controls 140 to 205 cells) (**Figures 5B, C**). The population II (patient 23%; controls 15-19%) and the population III were only slightly affected. The population IV was high (patient 28%; controls 11-20%) and the population V was low (patient 2%; controls 4-8%) (**Figure 5C**). The CXCR3 and CCR6 expressions were reduced in patient cells (populations II, III and IV) (**Figure 5C**). CCR7 was slightly reduced in II-IV Treg populations. In population V the CCR6 expression was high (patient 18%; controls 0-4,1%). The CD25 expression in CD4^+^ T cells was increased with a 2.2-fold increase in MFI (patient 510; control 237). 61% of the CD4^+^ T cells were positive for CD25 (controls 23-40%).

### 3.9 Transcription of inflammatory pathway genes in KLF2 variant patients

**Figure 6:**
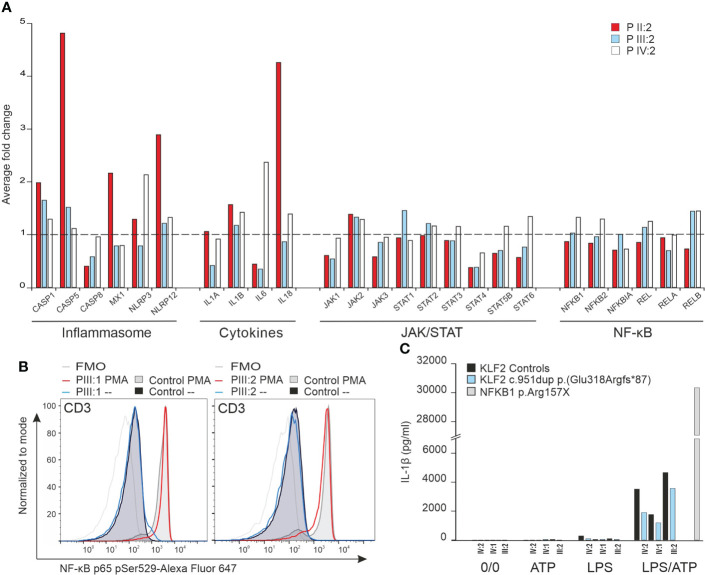
**(A)** NanoString analysis of inflammation related genes including NF-κB pathway, JAK/STAT pathway, cytokines and inflammasome was performed for mRNA from unstimulated PBMCs of three mutation carriers (IV:2, III:2 and II:2) and age/sex-matched controls. The average fold change in gene expression of inflammation related genes is illustrated. **(B)** NF-κB p65 Ser529 phosphorylation in CD3 lymphocytes. The NF-κB p65 Ser529 phosphorylation was measured by flow cytometry. Two mutation carriers (III:1 and III:2) and sex-matched controls´ PBMCs were unstimulated or stimulated with PMA or LPS for 15 minutes *in vitro* and stained for NF-κB p65 and CD3 markers. **(C)** Inflammasome activity assay. Mutation carrier and sex/age-matched control PBMCs were either unstimulated, ATP-stimulated, LPS-stimulated or LPS/ATP stimulated *in vitro* for 6 hours and 45 minutes (ATP added at 6 hours). The IL-1β production was measured from medium with ELISA. PBMCs from an autoinflammatory patient caused by NFKB1 p.Arg157X mutation were used for comparison to demonstrate uncontrolled IL-1β production.

KLF2 is an important regulator of the NF-κβ pathway (2). We performed NanoString analysis to explore the effects of the KLF2 p.(Glu318Argfs*87) variant on the transcription of genes commonly associated with the NF-κβ and other related immunological pathways. Transcription of genes related with NF-κβ pathway were comparable to age matched controls (**Figure 6A**). In addition, the JAK/STAT pathway was not compromised. Also, transcription of genes involved in regulation of NLRP3 inflammasome were like that of the controls (**Figure 6A**) although some activation (patient II:2) was seen. Transcription of genes related with interferon response were comparable to controls and no evidence of impaired interferon signature was detected (data not shown).

### 3.10 RelA/p65 and inflammasome activity

Arthritis in KLF2 deficient animal model is dependent on IL-1β (44) which in turn can be controlled by the NF-κβ pathway (2). Patients’ PBMCs stimulated with PMA and LPS, however, showed equal RelA/p65 phosphorylation compared to healthy controls (**Figure 6B**). IL-1β production after activation of monocytes with ATP/LPS was also comparable to healthy controls (**Figure 6C**). These data suggest that heterozygosity for the KLF2 p.(Glu318Argfs*87) variant does not harm the regulation of the NF-κβ pathway least under *in vitro* conditions.

## 4 Discussion

The novel c.952dup, p.(Glu318Argfs*87) variant disrupts the highly conserved zinc finger structures including the amino acids that are most essential for the activity of KLF transcription factor family (34). For example, all three zinc finger structures are needed for efficient nuclear localization and DNA binding (32). In a previously published experimental model, the nuclear entry was approximately 50% when the first loop was intact and the second and third zinc finger loops were deleted (32). Consistent with the previous findings, we showed that this novel KLF2 variant disturbs the nuclear entry. In addition, we demonstrated a reduction in the wild type KLF2 protein in c.952dup neutrophils compared to controls. Although the mutant KLF2 protein cannot be quantitated with western blot, we cannot exclude its presence in low amounts. It is noteworthy that only limited numbers of uniallelic variants, especially in exonic regions, have been described in public databases. Examination of the DNA binding zinc finger domains (amino acids 272-296; 302-326; and 332-354) reveals 18 missense variants in gnomAD, of these 15 are detected with allele count 1 and the three others with allele counts 2,3 and 4. The AlphaFold model for the KLF2 zinc finger domains shows that all these variant amino acids are not involved in the DNA binding. In addition, in the proximal splice region or intron region of the zinc finger domains 51 nucleotide variations are detected, however only one variant (19-16436852-C-T, with allele count 3) was predicted to have a probable change in splicing (Donor gain 0.62, -2 bp) by SpliceAI (PMID: 30661751). However, four other frameshift variants, resulting in a complete or partial loss of the zinc finger domains, exists in gnomAD, all detected with allele count of 1. These five variants can thus possibly affect the DNA binding domain in ways that may not differ significantly from the detected p.(Glu318Argfs*87) variant, and would warrant a follow-up examination outside this article.

The protein-protein interactions of the wild-type KLF2 are incompletely understood. For example, several wild-type KLF2 interactions observed in our analysis are implicated in immune responses. The mutant KLF2 expressed in HEK293 cells disrupts the interactome. The mutant loses the interactions with proteins linked to several pathways related to immune regulation whereas the pathways related to cell cycle were enriched. Interestingly, cyclin-dependent kinase 4, with high importance in cancer immunity (45), was the highest abundant interaction among the novel, gained interactions of the mutant KLF2. In addition, several additional targets of lost or gained interactions caused by the mutation are associated with malignancy or central nervous system biology. The interactome analysis shows that the KLF2 variant zinc finger domain disturbs the protein binding properties of the N-terminus. It is also evident that the nuclear localization and DNA binding properties are affected by the variant. Based on these results, this uniallelic KLF2 variant may exert gain-of- or dominant negative or both functions to downstream signalling, and as such requires further studies. We emphasize that the interactome experiments were conducted in a cell line, and we were not able to confirm the presence of the variant KLF2 gene product in patient’s cells. There are no antibodies available that could differentiate between the variant and the wildtype KLF2.

KLF2 may be of importance in the biology of malignant cells with tumor suppressive properties (46). Three of the family members (I:1, II:1, II:2) developed malignancies and one family member (II:2) two separate cancers. Somatic KLF2 mutations involving the zinc finger domain are commonly found in splenic marginal zone B cell lymphoma (SMZL) (15) although their role is incompletely understood (17, 18). KLF2 mutation is present in 42% of SMZLs, but rarely in other B-cell lymphomas (17). One variant positive family member (II:1) experienced a transformation of chronic lymphocytic leukemia (CLL) into fast growing treatment resistant B-cell lymphoma at a young age. We conclude that a possibility of lymphoma should be considered in patients with KLF2 genetic variant.

KLF2 deficiency in animal models results in an increased number of marginal zone (MZ) cells, decreased B1 B cells and low serum IgG1 (47). The KLF2 variant positive cases have low B cells, switched memory B cells and serum IgG1. The B cell abnormalities are comparable to those of common variable immunodeficiency (CVID) caused by deficiency of NFKB1 (48) which is in part regulated by KLF2 (2). However, our studies on p65/RelA phosphorylation, inflammasome activation and mRNA expression in mononuclear cells did not support the view that the KLF2 variant causes marked NF-κβ signaling imbalance. Although no serious effects on humoral immune deficiency were observed in experimental KLF2 deficiency models (38), the index patient (VI:2) with KLF2 variant fulfills the criteria for CVID (41). In addition to B cell deficiency in VI:2, variant positive cases displayed low peripheral blood T cell counts. Especially naive T cell and CD4^+^ effector memory T cell counts were low. L-selectin (CD62L) expression, which was reduced in the patients, is directly regulated by KLF2 (3). S1PR1 and CCR7 are also regulated by KLF2; while the total number of circulating KLF2 and S1PR1 positive T cells were very low in the KLF2 variant patients, expression of target genes among the rare peripheral blood T cells was only mildly affected. Low levels of these trafficking molecules, which are mainly expressed in peripheral tissues during inflammation, may at least partly explain the peripheral blood lymphopenia (49).

KLF2 is associated with development of autoimmunity and arthritis (44). Consistent with previous observations (39), the KLF2 variant carriers had an elevated percentage of CD38^low^CD21^low^ activated B cells. The patients displayed disturbed Treg development, associated with autoimmunity (50) and KLF2 deficiency in experimental models (51). Within the T cell compartment, the Treg cells were significantly affected by the KLF2 mutation: while the percentage of CD25^+^FOXP3^+^ and CD25^+^CD127^-^ was increased in the patients, the naive CD45RA^+^FOXP3^+^ Treg population was completely absent in the peripheral blood. These results are in agreement with previous understanding of KLF2 in Treg production and migration (7, 51). We postulate that KLF2 deficient Tregs in the patients may be unable to migrate to secondary lymphoid organs (51). Furthermore, arthritis in KLF2 deficient animals is dependent on IL-1β. Although our experiments did not suggest excessive inflammasome activation or increased IL-1β production, the NanoString analysis demonstrated a trend towards moderately increased transcription of inflammasome-related genes. We can only speculate that the disturbed Treg mediated peripheral tolerance would play an important role in the development of autoimmunity in the patients. Arthritis in patients II:1 and IV:2 was resistant to several antirheumatic therapies. However, patient IV:2 responded to anti-TNF drug etanercept at least partially. Patient II:1 did not receive biologicals because of malignant disease; it is not known whether he would have responded to anti-TNF medication.

In summary, we describe a family with a novel KLF2 mutation disrupting the highly conserved zinc finger domain needed for nuclear localization and DNA binding. The affected family members presented with lymphopenia and immune abnormalities, without discernible lung pathology. Some family members have remained asymptomatic for years suggesting variable expressivity and penetrance. Studies on additional KLF2 deficient families will be needed to understand the spectrum of clinical consequences. However, we believe that role of KLF2 gene should be considered whenever unexplained familial lymphopenia in association with autoimmunity and an increased risk of malignancy is encountered.

## Data availability statement

The datasets presented in this study can be found in online repositories. The names of the repository/repositories and accession number(s) can be found below: National Center for Biotechnology Information (NCBI) BioProject database under accession number SCV002588747.

## Ethics statement

The studies involving human participants were reviewed and approved by Oulu University Hospital. Written informed consent to participate in this study was provided by the participants’ legal guardian/next of kin. Written informed consent was obtained from the individual(s), and minor(s)’ legal guardian/next of kin, for the publication of any potentially identifiable images or data included in this article.

## Author contributions

NP, SK, IC, AN, VG, WS, SS, MV, PÅ: Immunological analysis, study design, data and manuscript preparation. RK-F: Genetic analysis. JJ: hemodynamic and cardiological evaluation. PV, TH: study design, clinical care, manuscript preparation. KE, MS: study design, manuscript preparation. All authors contributed to the article and approved the submitted version.

## Funding

This study was supported by Oulu University Hospital VTR (K74809).

## Conflict of interest

TH has received support from CSL-Behring.

The remaining authors declare that the research was conducted in the absence of any commercial or financial relationships that could be construed as a potential conflict of interest.

## Publisher’s note

All claims expressed in this article are solely those of the authors and do not necessarily represent those of their affiliated organizations, or those of the publisher, the editors and the reviewers. Any product that may be evaluated in this article, or claim that may be made by its manufacturer, is not guaranteed or endorsed by the publisher.
