# Supplementary material for: Heterozygous premature termination in zinc-finger domain of Krüppel-like factor 2 gene associates with dysregulated immunity
Source: Front Immunol. 2022 Nov 18;13:819929. doi: 10.3389/fimmu.2022.819929 (PMC9716311; doi:10.3389/fimmu.2022.819929)

Pernaa et al. Supplementary material.
Original images of western blots.

Proteins extracted from fibroblasts.

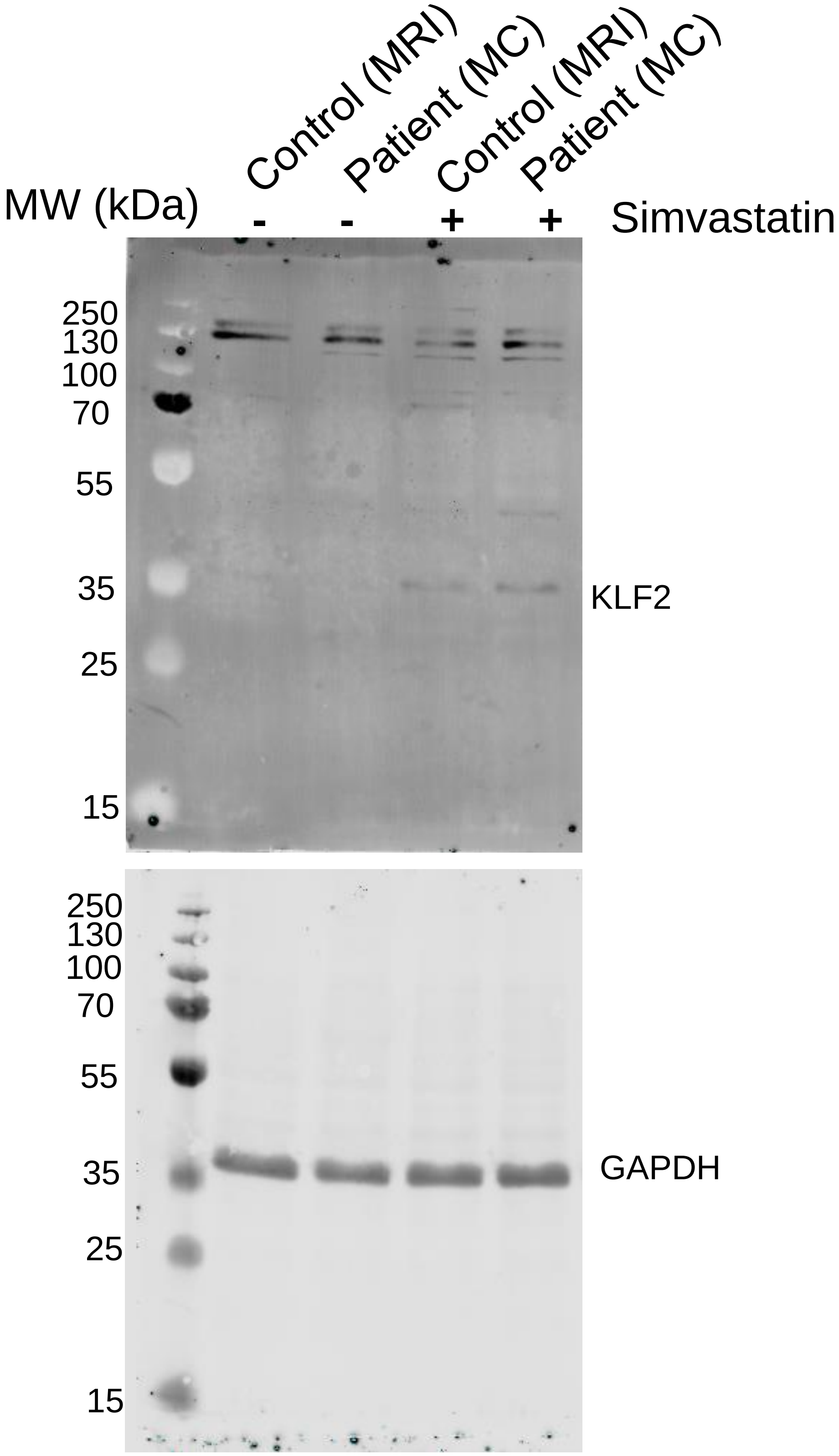

Proteins extracted from neutrophils (samples analysed as triplicates to measure differences in intensities)

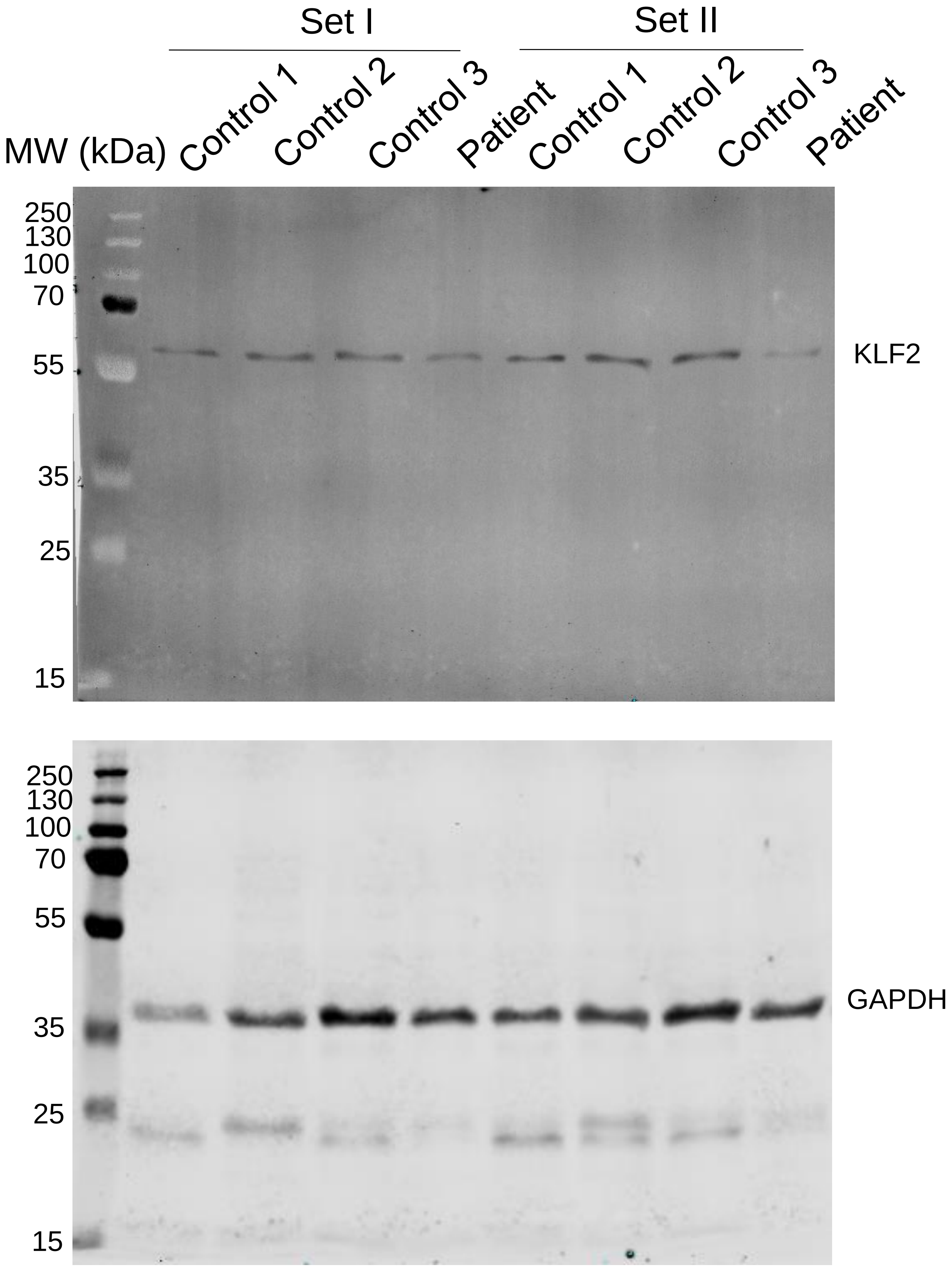

Proteins extracted from neutrophils

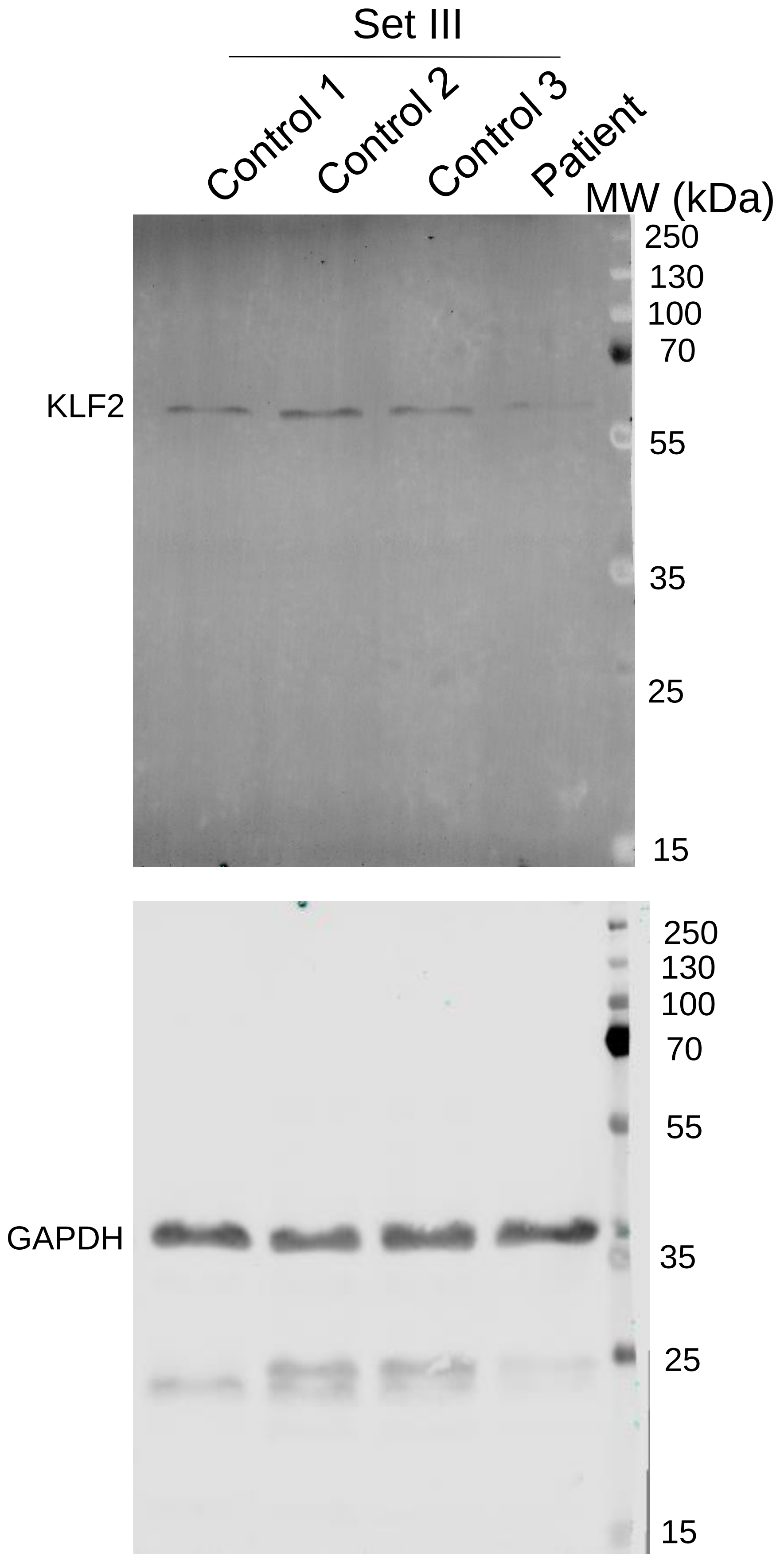

Supplement: Supplementary file 1 [file DataSheet_1.pdf]
