# Supplementary material for: Heterozygous premature termination in zinc-finger domain of Krüppel-like factor 2 gene associates with dysregulated immunity
Source: Front Immunol. 2022 Nov 18;13:819929. doi: 10.3389/fimmu.2022.819929 (PMC9716311; doi:10.3389/fimmu.2022.819929)
Supplement: Supplementary file 2 [file DataSheet_2.pdf]

SOURCE   Homo sapiens (human)

ORGANISM Homo sapiens

Eukaryota; Metazoa; Chordata; Craniata; Vertebrata; Euteleostomi;

Mammalia; Eutheria; Euarchontoglires; Primates; Haplorrhini;

Catarrhini; Hominidae; Homo.

REFERENCE   1 (bases 1 to 1068)

AUTHORS   Pernaa,N.

TITLE   Heterozygous premature termination in zinc-finger domain of

Kruppel-like factor 2 gene associates with dysregulated immunity

JOURNAL   Frontiers in immunology (2022) In press

REFERENCE   2 (bases 1 to 1068)

AUTHORS   Pernaa,N.

TITLE   Direct Submission

JOURNAL   Submitted (31-OCT-2022) Research Unit of Biomedicine, University of

Oulu, Aapistie 5A, Oulu, Suomi 90014, Suomi

COMMENT   Bankit Comment: ALT EMAIL:timo.hautala@oulu.fi

Bankit Comment: TOTAL # OF SEQS:1

##Assembly-Data-START##

Sequencing Technology :: Sanger dideoxy sequencing

##Assembly-Data-END##

FEATURES   Location/Qualifiers

source   1..1068

/organism="Homo sapiens"

/mol_type="mRNA"

/db_xref="taxon:9606"

/genotype="Disease causing variant"

gene   <1..1068

/gene="Human Kruppel like factor 2"

/allele="disease causing variant"

/note="DOI 10.3389/fimmu.2022.819929"

CDS <1..1068

/gene="Human Kruppel like factor 2"

/allele="disease causing variant"

/note="Human KLF2 disease causing variant"

/codon_start=1

/product="KLF2"

/translation="MALSEPILPSFSTFASPCRERGLQERWPRAEPESGGTDDDLNSV

LDFILSMGLDGLGAEAAPEPPPPPPPPAFYYPEPGAPPPYSAPAGGLVSELLRPELDA

PLGPALHGRFLLAPPGRLVKAEPPEADGGGGYGCAPGLTRGPRGLKREGAPGPAASCM

RGPGGRPPPPPDTPPLSPDGPARLPAPGPRASFPPPFGGPGFGAPGPGLHYAPPAPPA

FGLFDDAAAAAAALGLAPPAARGLLTPPASPLELLEAKPKRGRRSWPRKRTATHTCSY

AGCGKTYTKSSHLKAHLRTHTGEKPYHCNWDGCGWKFARSDELTRHYRKHTGHRPFQC

HLCDRAFSRSDHLALHMKRHM"

BASE COUNT 132 a 453 c 341 g 142 t

ORIGIN

1 atggcgctga gtgaacccat cctgccgtcc ttctccactt tcgccagccc gtgccgcgag

61 cgcggcctgc aggagcgctg gccgcgcgcc gaacccgagt ccggcggcac cgacgacgac

121 ctcaacagcg tgctggactt catcctgtcc atggggctgg atggcctggg cgccgaggcc

181 gccccggagc cgccgccgcc gcccccgccg cctgcgttct attaccccga acccggcgcg

241 cccccgccct acagcgcccc cgcgggtggc ctggtgtctg agctgctgcg acccgagctg

301 gatgcgccgc tggggcccgc actgcacggc cgctttctgc tggcgccgcc cggccgcctg

361 gtcaaggccg agcccctga agcggacggc ggcggcggct acggctgcgc ccccgggctg

421 acccgtggac cgcgcggcct caagcgcgag ggcgccccgg gcccggcggc ttcgtgcatg

481 cgaggtcccg ggggccgccc cccgccgccg cccgacacac cgccgctcag ccccgacggc

541 cccgcgcgcc tgcccgcgcc cggtccgcgc gcctccttcc cgccgccttt cggtggccct

601 ggtttcggcg cgcccgggcc cggcctgcat tacgcgccgc ctgcgccccc agccttcggt

661 ctcttcgacg acgcggccgc cgccgcggca gccctgggcc tggcgccccc cgccgcccgc

721 ggtctcctca cgccgcctgc gtccccgctg gagctgctgg aggccaagcc aaagcgcggc

781 cgccgctctt ggccccgcaa acgcaccgcc actcacacct gcagctacgc gggctgcggc

841 aagacctaca ccaagagttc gcatctgaag gcgcatctgc gcacgcacac aggtgagaag

901 ccctaccact gcaactggga cggctgcggc tggaagtttg cgcgctcaga cgagctcacg

961 cgccactacc gaaagcacac gggccaccgg ccattccagt gccatctgtg cgatcgtgcc

1021 ttctcgcgct ccgatcacct ggcgctgcac atgaaacggc acatgtag

//
